# Larvicidal Activity against* Aedes aegypti* and Chemical Characterization of the Inflorescences of* Tagetes patula*

**DOI:** 10.1155/2017/9602368

**Published:** 2017-12-07

**Authors:** Letícia Maria Krzyzaniak, Tânia Mara Antonelli-Ushirobira, Gean Panizzon, Ana Luiza Sereia, José Roberto Pinto de Souza, João Antonio Cyrino Zequi, Cláudio Roberto Novello, Gisely Cristiny Lopes, Daniela Cristina de Medeiros, Denise Brentan Silva, Eneri Vieira de Souza Leite-Mello, João Carlos Palazzo de Mello

**Affiliations:** ^1^Programa de Pós-Graduação em Ciências Farmacêuticas, Department of Pharmacy, Laboratory of Pharmaceutical Biology (Palafito), Universidade Estadual de Maringá, Avenida Colombo 5790, Maringá, PR, Brazil; ^2^Programa de Pós-Graduação em Agronomia, Department of Agronomy, Universidade Estadual de Londrina, Rodovia Celso Garcia Cid, Km 380, s/n, Londrina, PR, Brazil; ^3^Department of Animal and Plant Biology, Universidade Estadual de Londrina, Rodovia Celso Garcia Cid, Km 380, s/n, Londrina, PR, Brazil; ^4^Academic Department of Chemistry and Biology, Universidade Tecnológica Federal do Paraná, Linha Santa Bárbara, s/n, Francisco Beltrão, PR, Brazil; ^5^Laboratório de Produtos Naturais e Espectrometria de Massas (LAPNEM), Universidade Federal de Mato Grosso do Sul, Avenida Costa e Silva, s/n, Campo Grande, MS, Brazil; ^6^Department of Morphological Sciences, Universidade Estadual de Maringá, Avenida Colombo 5790, Maringá, PR, Brazil

## Abstract

The crude acetone extract (CAE) of defatted inflorescences of* Tagetes patula* was partitioned into five semipurified fractions:* n*-hexane (HF), dichloromethane (DF), ethyl acetate (EAF),* n*-butanol (BF), and aqueous (AQF). BF was fractionated by reversed-phase polyamide column chromatography, obtaining 34 subfractions, which were subjected to HSCCC, where patuletin and patulitrin were isolated. CAE and the fractions BF, EAF, DF, and AQF were analyzed by LC-DAD-MS, and patuletin and patulitrin were determined as the major substances in EAF and BF, respectively. BF was also analyzed by HPLC and capillary electrophoresis (CE), and patulitrin was again determined to be the main substance in this fraction. CAE and the semipurified fractions (750, 500, 300, 100, and 50 mg/L) were assayed for larvicidal activity against* Aedes aegypti*, with mortality rate expressed as percentage. All fractions except AQF showed insecticidal activity after 24 h exposure of larvae to the highest concentration. However, EAF showed the highest activity with more than 50% reduction in larval population at 50 mg/L. The insecticidal activity observed with EAF might have been due to the higher concentration of patuletin present in this fraction.

## 1. Introduction

*Aedes aegypti* (Linnaeus, 1762) is an anthropophilic and domicile mosquito, and it is the main vector for dengue viruses in the Americas. This mosquito puts half of the world's population at risk with a 30-fold increase in incidence in the past 50 years in more than 100 endemic countries [1, 2]. According to data from the World Health Organization, the number of people affected with dengue in 2015 was 3.2 million, with 500,000 people hospitalized per year [3].

*Ae. aegypti *also carries chikungunya, zika, and yellow fever urban viruses; so its monitoring and control are necessary. Vector control in Brazil currently occurs with the use of growth regulators of immature stages, such as diflubenzuron, and the control of adult mosquitoes with alpha-cypermethrin, deltamethrin, malathion, and others according to recommendations of the WHO Pesticide Evaluation Scheme [4], which are nonspecific products that select resistant insects due to their great genetic plasticity [5], with consequent environmental contamination [6].

There is currently a great deal of interest in alternative methods and selective principles for the control of mosquitoes with less environmental damage [7]. In this sense, substances extracted from plants present a great perspective for the control of* Ae. aegypti*.

The substances of natural origin have some advantages: they are obtained from renewable resources, and the selection of resistant forms occurs at a slower rate than with synthetic insecticides [8, 9]. Another advantage is that they show low or no toxicity to mammals and bees [10].

Among the plants with bioactive substances, there is* Tagetes patula* L., popularly known as “cravo-francês,” “cravo-de-defunto,” or “botões-de-solteirão” [11].* T. patula* belongs to the family Asteraceae, which is one of the oldest groups of higher plants [12], with approximately 300 genera and 3000 species in Brazil [13], and its flavonoids patuletin and patulitrin are considered important taxonomic markers [14].

Its inflorescences have been used in folk medicine for antiseptic, diuretic, blood purifying, and insect repellent purposes. Its leaves have been used for renal problems and muscle pain and its roots and seeds used as purgatives [15]. Some studies on the chemical composition of* T. patula* up to now indicate that the flowers and leaves are rich in terpenes [16, 17], alkaloids [18], thiophenes [19], and flavonoids [20–22]. This plant has shown the following activities: antihypertensive [23], anti-inflammatory [14], hepatoprotective [24], insecticidal [25], nematicidal [26, 27], larvicidal [19], antibacterial [17], antiviral [28], and antifungal [29].

Accordingly, the aim of this work was to isolate and identify compounds from the semipurified* n*-butanol fraction of* T. patula* by reversed-phase column chromatography and high-speed countercurrent chromatography (HSCCC) and to evaluate the chemical profile of the crude extract and semipurified fractions using high performance liquid chromatography (HPLC), capillary electrophoresis (CE), and liquid chromatography-mass spectrometry (LC-DAD-MS). In addition, the larvicidal activity of the crude extract and semipurified fractions was evaluated against* Ae. aegypti*.

## 2. Materials and Methods

### 2.1. Plant Material

The inflorescences of* T. patula* were collected in November 2011 in the Garden of Medicinal Plants of the Universidade Estadual de Londrina, Londrina, Brazil, where they were organically grown. The plant material was collected under a permit from IBAMA-SISBIO, number 11995-6, May 13, 2016, authentication code 48926652, under the responsibility of J. C. P. Mello. An exsiccate is deposited at the Herbarium of the Universidade Estadual de Maringá (HUEM) under number 21907, and the identification was provided by Professor Dr. Jimi Nakajima at the Institute of Biology of the Universidade Federal de Uberlândia, Uberlândia, Brazil. The flowers were dried in a convection oven at 38°C for 48 h. The dried plant material was macerated using a hammer mill (Tigre ASN-5).

### 2.2. Preparation of Crude Extract and Semipurified Fractions

The milled inflorescences (1.9 kg) were defatted with* n*-hexane by dynamic maceration for three days, with subsequent drying of the inflorescences at room temperature. Afterwards, acetone was used as extraction solvent at a proportion of 4% (w/v) in an Ultra-Turrax® (UTC115KT, Ika Works) for 5 min and then subjected to maceration for 15 h. Next, turbo-extraction was performed for 20 min, with intervals of 5 min (*t* < 40°C). The extract was filtered, concentrated under reduced pressure, frozen, and lyophilized (Alpha 1–4, Christ®) to give the crude acetone extract (CAE, 5.86%). CAE was fractionated according to Filho and Yunes [30]. Briefly, 105 g CAE was resuspended in 1 L of methanol : water (2 : 8, v/v) and partitioned with different solvent volume ratios. The yields were* n*-hexane (HF) 19.27%, dichloromethane (DF) 10.17%, ethyl acetate (EAF) 13.38%,* n*-butanol (BF) 36.59%, and aqueous (AQF) 15.02%.

### 2.3. Reversed-Phase Column Chromatography of* n*-Butanol Fraction

BF (20.0 g) was separated by column chromatography (CC) with a polyamide column (CC6 Korngrobe, 0.05–0.16 mm; Macherey Nagel) according to Degani et al. [31], and the mobile phase was 100% methanol or water or a combination thereof, providing 34 subfractions (BF#1–34). The subfractions BF#6 (25 mg) and BF#11 (5 mg) precipitated during the organic solvent removal process and were analyzed by nuclear magnetic resonance (NMR), MS, and HPLC.

### 2.4. High-Speed Countercurrent Chromatography (HSCCC)

The subfractions BF#16, BF#23, and BF#26 were rechromatographed by HSCCC using a PC Ito® chromatograph (model 001) equipped with a polytetrafluoroethylene (PTFE) column (2.5 mm i.d., total volume capacity of 320 mL), 10-*μ*L sample loop, 800 rpm, and double piston solvent pump (Waters model 510), using a flow-rate of 1.0 mL/min. The organic phase (hexane : ethyl acetate : methanol : water; Table 1) was used as the mobile phase, and water was the stationary phase. Only in HSCCC of BF#16 was a gradient system with* n*-butanol also used. BF#16, BF#23, and BF#26 yielded 8, 15, and 18 subfractions, respectively. The subfractions BF#16.5 (7 mg), BF#23.4 (13 mg), BF#26.6 (4.5 mg), and BF#26.15 (5 mg) were selected and analyzed by NMR and MS.

### 2.5. NMR Analysis

The subfractions BF#6, BF#11, BF#16.5, BF#23.4, BF#26.6, and BF#26.15 were analyzed by NMR spectroscopic methods 1D (^1^H and ^13^C) and 2D (^1^H/^1^H-COSY and HMBC), with a Varian Mercury Plus 300 (75 MHz for ^13^C and 300 MHz for ^1^H), using deuterated solvents and TMS as internal reference. The spectra of the subfractions were related to the compounds** Tp1** (BF#23.4, #26.6, and #26.15) and** Tp2** (BF#6, #11, and #16.5), which were analyzed and compared to literature data.

*Patuletin (* ***Tp1***) ^1^*H-NMR (CD*_3_*OD, 300 MHz)*. 6.48 (H-8), 7.72 (d,* J* 2.0 Hz, H-2′), 6.88 (d,* J* 8.08 Hz, H-5′), 7.63 (dd,* J* 8.6 Hz; 2.0 Hz, H-6′), 3.88 (OCH_3_-6). ^13^C-NMR (CD_3_OD, 75 MHz): 146.9 (C-2), 135.6 (C-3), 176.2 (C-4), 151.6 (C-5), 130.8 (C-6), 157.1 (C-7), 93.4 (C-8), 152.3 (C-9), 103.5 (C-10), 120.3 (C-1′), 114.6 (C-2′), 144.8 (C-3′), 147.4 (C-4′), 114.8 (C-5′), 122.8 (C-6′), 59.60 (CH3-6).

*Patulitrin (* ***Tp2***) ^1^*H-NMR (CD*_3_*OD, 300 MHz)*. 6.93 (s) (H-8), 7.72 (d,* J* 2.2 Hz, H-2′), 6.89 (d,* J* 8.5 Hz, H-5′), 7.54 (dd,* J* 8.5 Hz; 2.1 Hz, H-6′), 5.13 (d;* J* 7.2, H-1′′), 3.32 (d;* J* 2,2, H-2′′) 3.45 (m) (H-3′′), 3.17 (m) (H-4′′), 3.48 (m) (H-5′′), 3.72 (m) (H-6′′), 3.78 (s) CH_3_O-6. ^13^C-NMR (CD_3_OD, 75 MHz): 147.9 (C-2), 135.8 (C-3), 176.2 (C-4), 151.1 (C-5), 131.8 (C-6), 156.4 (C-7), 93.8 (C-8), 151.4 (C-9), 105.0 (C-10), 120.0 (C-1′), 115.5 (C-2′), 145.0 (C3′), 147.7 (C-4′), 115.4 (C-5′), 121.8 (C-6′), 100.1 (C-1′′), 73.2 (C-2′′), 77.2 (C-3′′), 69.5 (C-4′′), 76.7 (C-5′′), 60.6 (C-6′′), 60.3 (CH_3_O-6).

### 2.6. HPLC-ESI-MS/MS Analysis

Fractions and subfractions were analyzed with a Waters HPLC system coupled with a triple quadrupole mass spectrometer (Micromass, Quattro micro™ API) equipped with a Z-electrospray ionization (ESI) source (Waters) and processed by MassLynx™ software (version 4.0, Waters). Chromatographic conditions were as follows: column was a Symmetry C-18 (3.5 *μ*m, 75 × 4.6 mm, Waters); mobile phase was water with 0.1% formic acid (v/v) (solvent A) and acetonitrile with 0.1% formic acid (v/v) (solvent B). The gradient system employed was as follows: 0–2 min 5% B; 10 min 50% B; 2 min 50%; and 13–15 min 5% B. The flow rate was 0.5 mL/min and the injection volume 10 *μ*L. A sample containing 1000 ng/mL of the isolated substances was injected, and identification was performed analyzing the information of the product ion spectra in comparison to a previously published dataset.

### 2.7. Identification of the Constituents by LC-DAD-MS

The analyses of CAE and the fractions DF, EAF, BF, and AQF were performed on UFLC Shimadzu Prominence chromatograph coupled to a diode array detector (DAD) and MicrOTOF-Q III mass spectrometer (Bruker Daltonics). A Kinetex C-18 chromatographic column (2.6 *μ*m, 150 × 2.1 mm, Phenomenex) was used. Acetonitrile (solvent B) and deionized water (solvent A), both with 0.1% formic acid (v/v), were used as mobile phase. The gradient elution profile was the following: initial 3% B, 2–25 min 25% B, 25–40 min 80% B, and 40–43 min 80% B. The negative and positive ion modes were carried out, and nitrogen was applied as a nebulizer gas (4 bar) and dry gas (9 L/min).

### 2.8. Capillary Electrophoresis (CE)

CE for BF analysis was carried out using a Beckman P/ACE™ MDQ electrophoresis system equipped with a filter-based UV/Vis detector and 32 Karat™ version 7.0 software. The column used was a fused silica capillary column (Beckman-Coulter) with dimensions of 60.2 cm total length, 50.0 cm effective length, 363 *μ*m o.d., and 75 *μ*m i.d. The sample was injected hydrodynamically at 0.5 psi for 3 s, 30 kV, and the electropherogram was recorded at 214 nm. The cartridge coolant of the CE was set with a thermostat at 25°C. The background electrolyte consisted of 80 mmol/L borate buffer (pH 8.80) containing 10 mmol/L methyl-*β*-cyclodextrin (Me-*β*-CD). The sample solution (500 *μ*g/mL) was prepared by dissolving 5 mg BF in 10 mL of 20% methanol and was eluted through the solid-phase extraction (SPE) cartridge (Strata C18-E, Phenomenex), preconditioned with methanol and water.** Tp1** and** Tp2** were used as standards for peak identification. All solutions were filtered with 0.45 *μ*m Millipore filters.

### 2.9. Evaluation of Larvicidal Activity

CAE, AQF, EAF, HF, BF, DF, and the fatty waste obtained in the preparation of the crude extract were tested for larvicidal activity.

Immature forms of* Ae. aegypti* were obtained from the insectary of the Malaria and Dengue Laboratory, Instituto Nacional de Pesquisas da Amazônia (INPA), Manaus, Brazil. The insectary began with the collection of eggs in the field by using traps (egg traps). All the procedures for the maintenance of mosquitoes and the use of animals for blood meal were authorized by the Animal Experiment Ethics Committee (CEUA/INPA 04/2013). The bioassay methods were according to Lacey [32], and WHO [33, 34], with modifications.

Fourth instar larvae were used for all experiments. Three replicates with 15 immature forms and 50 mL of distilled water per container were assayed. The crude extract and semipurified fractions were diluted in dimethyl sulfoxide (DMSO) at an initial concentration of 30,000 mg/L in a total volume of 10 mL. The samples were solubilized using an ultrasonic bath for 15 min. To determine mortality rates in percent, lethal concentrations (LC_50_ and LC_90_), and their limits, five concentrations were used: 750, 500, 300, 100, and 50 mg/L. DMSO solution at 300 mg/L and distilled water were used as controls. The assay was performed using a photoperiod of 12/12 h, at 26 ± 2°C. Mortality readings were performed at 24, 48, 72, 96, and 120 h.

Statistical package Spss Inc. 2005 was used for the calculation of the survival curve of the fractions and the lethal time of* Ae. aegypti *for EAF.

## 3. Results and Discussion

### 3.1. Structural Analysis

The structural analysis of subfractions BF#6, BF#11, BF#16.5, BF#23.4, BF#26.6, and BF#26.15 was performed by NMR, HPLC-IES-MS/MS and LC-DAD-MS and resulted in the identification of the compounds** Tp1** and** Tp2**.

**Tp1** (BF#23.4, BF#26.6, and BF#26.15) was obtained as a yellow powder. The mass spectrum obtained by ESI showed an intense ion peak at *m*/*z* 333, corresponding to the protonated ion and fragment ions of *m*/*z* 288 and 318. The UV spectrum of** Tp1** revealed two absorption maxima in the region of 257 nm (band I) and 372 nm (band II), compatible with the UV spectrum of flavonols.

**Tp2** (BF#6, BF#11, and BF#16.5) was also obtained as a yellow powder. Its mass spectrum showed an intense ion of *m*/*z* 495 and fragment ions at *m*/*z* 318 and 333. The UV spectrum of** Tp2** revealed maxima at 258 nm (band I) and 371 (band II), which was also compatible with flavonols.

On the basis of ^1^H and ^13^C-NMR data obtained and comparison with literature values [35–37],** Tp1** and** Tp2** were identified as the flavonols patuletin and patuletin-7-*O*-*β*-glycoside (patulitrin), respectively, which was confirmed by HMBC, HSQC, and COSY data.

**Figure 1:**
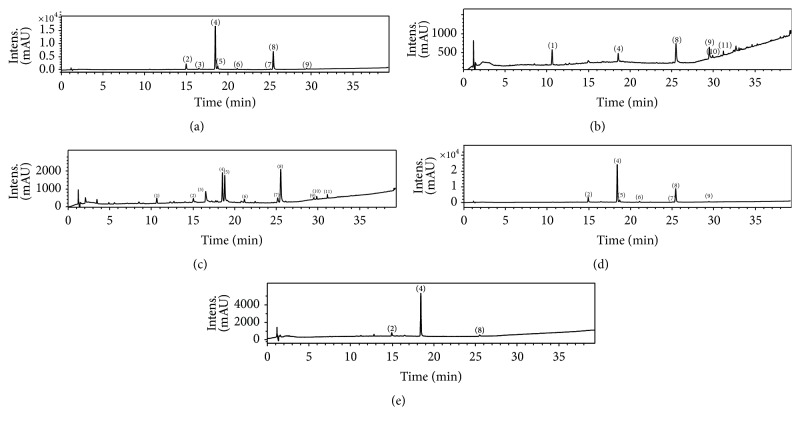
Chromatogram at 240 to 350 nm of the crude acetone extract of* Tagetes patula* (a) and its fractions obtained with dichloromethane (b), ethyl acetate (c),* n*-butanol (d), and water (e). The identification of the constituents is given in Table 2.

CAE and AQF, EAF, DF, and BF from* T. patula* were analyzed by LC-DAD-MS, and the compounds were identified on the basis of UV and accurate mass and fragmentation data, which were compared with the literature data. From the samples, eleven compounds were detected and identified (Figure 1, Table 2). The higher peak intensity was compound 4 (patulitrin) for BF and AQF, compounds 4, 5 (patulitrin isomer), and 8 (patuletin) for EAF, compound 8 for DF, and compounds 4 and 8 for CAE (Figure 1).

### 3.2. CE Fingerprint of BF of* T. patula*

**Figure 2:**
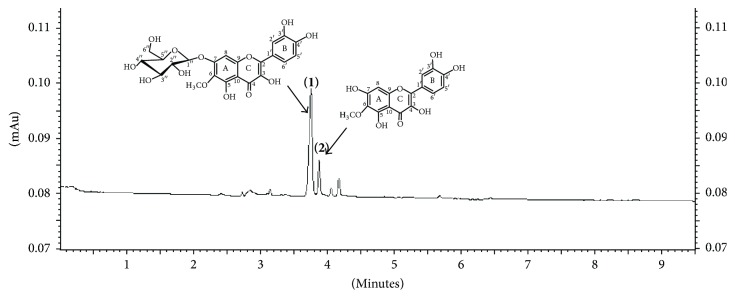
CE-UV electropherogram of the* n*-butanol fraction of* Tagetes patula*. Experimental conditions: 80 mmol/L borate buffer at pH 8.80 with 10 mmol/L Me-*β*-CD; uncoated fused-silica capillary column, 60.2 cm (50 cm effective length) × 75 *μ*m i.d.; 30 kV; 25°C; hydrodynamic injection 0.5 psi × 5 s; detection at 214 nm; BF: 500 *μ*g/mL. Peaks:** (1)  Tp2** (patulitrin); ** (2) Tp1** (patuletin).

In this work, BF of* T. patula* was evaluated by CE. The major peaks were identified by addition of the isolated substances of this work. Peak 1 was identified as** Tp2** and peak 2 as** Tp1** (Figure 2). This fingerprint shows that the major substance was** Tp2**, and the same profile was observed by LC-DAD-MS analysis (Figure 1).

Some studies with* T. patula* have been performed using thin layer chromatography (TLC), HPLC, and HPLC-MS [19, 38]. However, CE was employed here for the first time to identify the compounds obtained from* T. patula*.

Comparing the HPLC and CE methods developed for evaluation of BF of* T. patula*, CE was more efficient, being almost four times faster. In addition, in the CE method, organic solvents are not used to separate the analytes, and the volume of electrolytic solution used for analyses is small, making the technique less costly and polluting [39–41].

### 3.3. Larvicidal Activity

All the fractions of* T. patula* evaluated showed insecticidal activity against* Ae. aegypti* after 24 h exposure of the larvae to a concentration of 750 mg/L, with exception of AQF.

After 120 h (5 days) of exposure, the following mortality rates were observed at a concentration of 300 mg/L for the different samples evaluated: CAE (31.0%), AQF (17.8%), EAF (53.0%), HF (13.0%), BF (15.6%), DF (8.9%), and fatty waste (31.0%). No deaths occurred during a four-day observation period for the DMSO control, but at the end of the fifth day, mortality was 6.7%. The distilled water control did not cause any mortality during the whole experiment period (Table 3).

**Figure 3:**
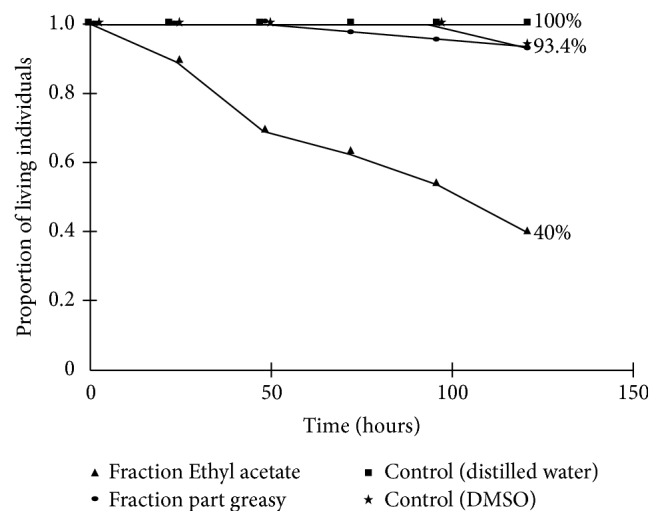
Survival rates (log-rank test) of the immature stages of* Ae. aegypti* exposed to controls (distilled water and DMSO), ethyl acetate fraction, and fatty waste of* Tagetes patula* at 50 mg/L, for 120 h (*p* < 0.0001; variance: 13.96; chi-square: 191.38).

EAF and fatty waste showed the best time-dependent results (Figure 3). The lethal time for 50 percent mortality (LT_50_) of* Ae. aegypti* with EAF was 96.7 h (range: 78.4–134.8 h).

Komalasmisra et al. [42] demonstrated that plants with a LC_50_ lower than 750 mg/L for larvicidal activity are effective against* Ae. aegypti*. Thus, CAE and all fractions evaluated, with the exception of AQF, showed notable larvicidal activity in the present study. Among the fractions analyzed, EAF was the most promising, where a concentration as low as 50 mg/L reduced the larval population by more than half, and where 750 mg/L caused the death of all larvae within 96 h.

Faizi et al. [27] carried out a nematicidal study with flowers of* T. patula*, which were first subjected to a defatting process with petroleum ether and then extracted with methanol, finally resulting in aqueous, dichloromethane, ethyl acetate, and butanol fractions. In that study, EAF had a higher concentration of patuletin, while the BF showed a lower amount of this substance. The authors reported that patuletin is generally more potent than patulitrin in other biological assays, such as antimicrobial and antioxidant.

Comparing our results of larvicidal activity against* Ae. aegypti* with those for nematicidal activity against* Heterodera zeae* reported by Faizi et al. [27], it is observed that, in both studies, the fraction with better activity was that with a higher concentration of patuletin and lower level of patulitrin. Thus, it is suggested that the larvicidal activity observed in EAF may be due to the higher concentration of patuletin seen in this fraction.

## 4. Conclusion

Among the semipurified fractions obtained from CAE of the inflorescences of* T. patula*, BF showed a higher yield of the flavonoids patuletin and patuletin-7-*O*-*β*-glycoside (patulitrin).

LC-DAD-MS analysis of CAE and the fractions DF, EAF, BF, and AQF confirmed that the main substance in EAF was patuletin and patulitrin in BF.

EAF showed the highest larvicidal activity against* Ae. aegypti *with more than 50% decrease in larval population at a concentration of 50 mg/L. This high insecticidal activity observed in EAF may be due to the higher concentration of patuletin in this fraction.

## Figures and Tables

**Table 1:** Eluent systems used for HSCCC to obtain subfractions.

Subfraction	Eluent systems (v/v)
FB#16	hexane : ethyl acetate : methanol : water (2 : 2 : 2.5 : 2) Gradient elution with *n*-butanol: 0–400 mL – 0 mL *n-*butanol 400–800 mL – 20 mL *n-*butanol 800–1200 mL – 30 mL *n-*butanol 1200–1400 mL – 40 mL *n-*butanol

FB#23	hexane : ethyl acetate : methanol : water (1 : 5 : 1 : 5)

FB#26	hexane : ethyl acetate : methanol : water (2 : 2 : 2.5 : 2)

**Table 2:** Identification of the constituents from *Tagetes patula* by LC-DAD-MS.

Peak	RT (min)	Compound	UV (nm)	MF	Negative mode (*m*/*z*)		Positive mode (*m*/*z*)	
					MS (^*∗*^)	MS/MS	MS (^*∗*^)	MS/MS
(1)	10.6	NI	274					
(2)	15.0	Quercetagetin *O*-hexoside	270, 355	C_21_H_20_O_13_	479.0822	317, 195, 167	481.0969	319, 273, 199, 181, 169
(3)	15.5	Ellagic acid^st^	290, 360	C_14_H_6_O_8_	300.9990	284, 245, 229	303.0117	285, 275, 257, 247
(4)	18.5	Patulitrin^st^	257, 369	C_22_H_22_O_13_	493.0978	331, 316, 287, 271, 181, 166	495.1142	333, 318, 301, 273
(5)	18.8	Patulitrin isomer	260, 351	C_22_H_22_O_13_	493.0975	330, 315, 287	495.1106	333, 318
(6)	21.2	Isorhamnetin *O*-hexoside	270, 360	C_22_H_22_O_12_	477.1041	314, 299, 271, 181, 166	479.1203	317, 302
(7)	25.2	Kaempferol	267, 345	C_15_H_10_O_6_	285.0391	175	287.0547	241, 161, 153
(8)	25.5	Patuletin^st^	257, 369	C_16_H_12_O_8_	331.0462	316, 287, 271, 181, 166	333.0613	318, 290, 273
(9)	29.5	*O*-Methyl kaempferol	270, 355	C_16_H_12_O_7_	315.0502	300, 271, 255, 243, 166	317.0655	302, 274, 257, 245, 169
(10)	29.9	Tricoumaroyl spermidine	299, 310	C_34_H_37_N_3_O_6_	582.2591	—	584.2775	438, 420, 292, 275, 218, 204, 146
(11)	31.2	Coumaroyl spermidine derivative	296, 306	C_41_H_50_N_6_O_10_	785.3517	—	787.3690	641, 623, 495, 477, 275, 204

RT: retention time; MF: molecular formula; ^*∗*^error lower than 8 ppm; ^st^confirmed by authentic standard.

**Table 3:** Percentage of mortality of *Aedes aegypti* larvae exposed to different fractions of *Tagetes patula* under laboratory conditions at 300 mg/L, for 120 h.

Sample	24 h	48 h	72 h	96 h	120 h
Crude acetone extract	0	4.4	22.0	26.7	31.0^B^
Fatty waste	0	4.4	16.0	24.4	31.0^B^
Aqueous fraction	0	0	0	0	17.8^B*∗*^
Ethyl acetate fraction	22.0	31.0	38.0	49.0	53.0^A^
*n*-Hexane fraction	2.0	4.4	4.4	4.4	13.0^B^
*n*-Butanol fraction	0	11.0	13.0	13.3	15.6^B^
Dichloromethane fraction	4.4	4.4	8.9	8.9	8.9^B^
DMSO	0	0	0	0	6.7^B^
Distilled water	0	0	0	0	0^B^

^*∗*^Numbers followed by same letters in a column do not differ according to Tukey test (*p* = 0.01).

## Supplementary Materials

Supplementary Materials Figure S1. High performance liquid chromatography fingerprint of CAE (A) and BF (B) from the inflorescences of* Tagetes patula.* Peaks: (1)** Tp2** (patulitrin) and (2)** Tp1** (patuletin).
