# Supplementary material for: Larvicidal Activity against Aedes aegypti and Chemical Characterization of the Inflorescences of Tagetes patula
Source: Evid Based Complement Alternat Med. 2017 Dec 7;2017:9602368. doi: 10.1155/2017/9602368 (PMC5738574; doi:10.1155/2017/9602368)
Supplement: Supplementary Materials — Figure S1. High performance liquid chromatography fingerprint of CAE (A) and BF (B) from the inflorescences of Tagetes patula. Peaks: (1) Tp2 (patulitrin) and (2) Tp1 (patuletin). [file 9602368.f1.pdf]

Supplementary Material

High performance liquid chromatography analysis (HPLC)

BF and its 34 subfractions were analyzed by HPLC according to the method described by Munhoz et al. (2012), using a Thermo® Chromatograph with a Finnigan Surveyor LC Pump Plus and autosampler equipped with a 10-μL injection loop. Data were acquired and processed by ChromQuest® software. The isolated and identified compounds patuletin (**Tp1**) and patuletin-7-*O*-β-glycoside (patulitrin [**Tp2**]) were used as standards.

HPLC results

The HPLC fingerprint of CAE revealed two peaks: (1) t_r; 16.9 min and (2) t_r; 23.7 min (Figure S1A), identified as **Tp2** and **Tp1**, respectively. The fingerprint of the semipurified fraction BF (Figure S1B) showed a major peak with t_r of 16.9 min, corresponding to compound **Tp2,** and a minor peak identified as **Tp1**.

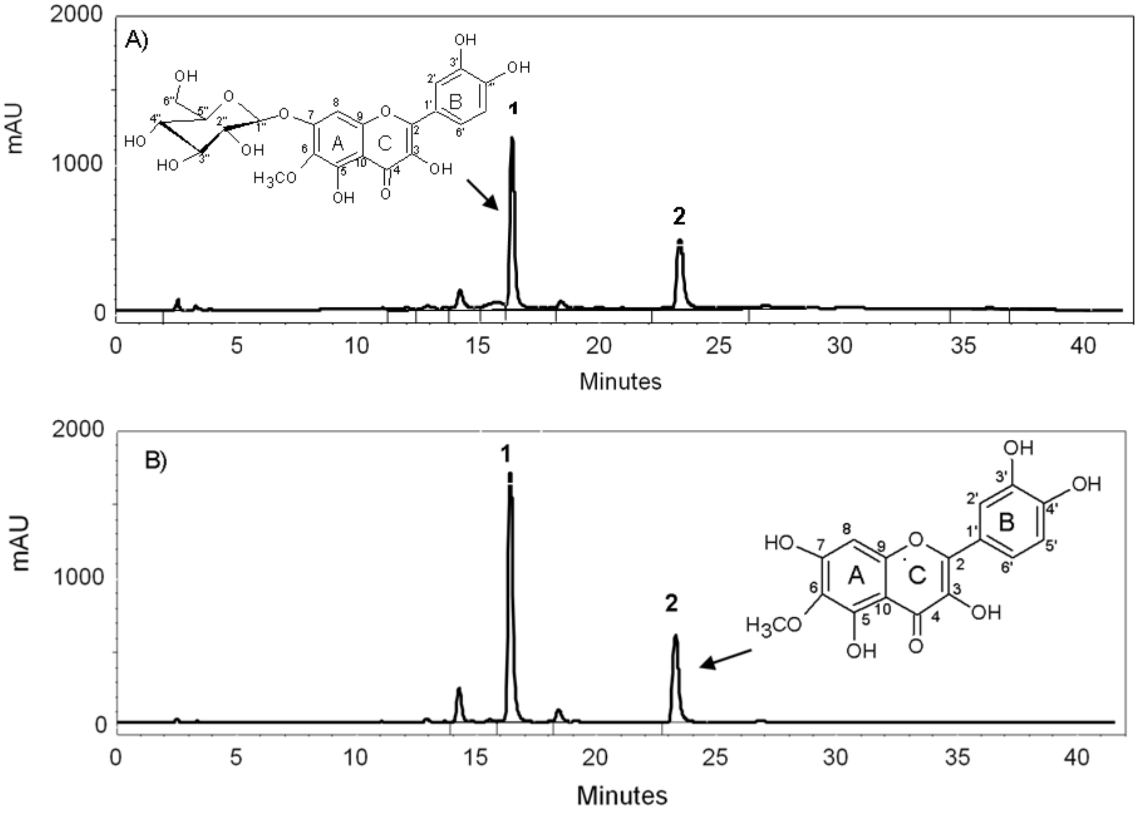

Figure S1. HPLC fingerprint of CAE (A) and BF (B) from the inflorescences of *Tagetes patula.* Peaks: 1) **Tp2** (patulitrin) and (2) **Tp1** (patuletin).
